# Mechanical Stretch on Human Skin Equivalents Increases the Epidermal Thickness and Develops the Basement Membrane

**DOI:** 10.1371/journal.pone.0141989

**Published:** 2015-11-03

**Authors:** Eijiro Tokuyama, Yusuke Nagai, Ken Takahashi, Yoshihiro Kimata, Keiji Naruse

**Affiliations:** 1 The Department of Plastic and Reconstructive Surgery, Okayama University Graduate School of Medicine, Okayama, Japan; 2 Menicon Co., Ltd., Aichi, Japan; 3 The Department of Cardiovascular Physiology, Okayama University Graduate School of Medicine, Dentistry and Pharmaceutical Sciences, Okayama, Japan; Université de Technologie de Compiègne, FRANCE

## Abstract

All previous reports concerning the effect of stretch on cultured skin cells dealt with experiments on epidermal keratinocytes or dermal fibroblasts alone. The aim of the present study was to develop a system that allows application of stretch stimuli to human skin equivalents (HSEs), prepared by coculturing of these two types of cells. In addition, this study aimed to analyze the effect of a stretch on keratinization of the epidermis and on the basement membrane. HSEs were prepared in a gutter-like structure created with a porous silicone sheet in a silicone chamber. After 5-day stimulation with stretching, HSEs were analyzed histologically and immunohistologically. Stretch-stimulated HSEs had a thicker epidermal layer and expressed significantly greater levels of laminin 5 and collagen IV/VII in the basal layer compared with HSEs not subjected to stretch stimulation. Transmission electron microscopy revealed that the structure of the basement membrane was more developed in HSEs subjected to stretching. Our model may be relevant for extrapolating the effect of a stretch on the skin in a state similar to an *in vivo* system. This experimental system may be useful for analysis of the effects of stretch stimuli on skin properties and wound healing and is also expected to be applicable to an *in vitro* model of a hypertrophic scar in the future.

## Introduction

In general, sites undergoing strong stretch stimulation such as articular extensors are known to have thickened skin, retarded wound healing, and increased incidence of hypertrophic scars and keloid. However, the causes are still not well understood.

The skin and other cells constituting our body are always exposed to some type of mechanical stimuli; for example, vascular endothelial cells are under sheer stress from blood flow and the vessel wall is under stretch stimulation associated with cardiac beat. Reports of experiments analyzing responses of cultured cells to stretch stimulation began to appear in 1970s [1], and to date, involve various types of cells, including myoblasts [2], vascular endothelial cells [3, 4], chondrocytes [5], and bronchial fibroblasts [6]. A stretch stimulus applied to cells triggers intracellular biochemical signaling, which is known to elicit cellular responses, such as increased expression of various proteins, altered gene expression, and differentiation and proliferation of the cells. In addition to 2-dimensional cultures, studies conducted in combination with 3-dimensional cultures have emerged in recent years [2, 7–9].

Many experiments involving stretch stimuli to cultured skin cells have also been reported [7, 8, 10–28]. To the best of our knowledge, all of these experiments were performed on either epidermal keratinocytes or dermal fibroblasts alone. With respect to fibroblasts, although experiments in 3-dimensional culture have also been published [7–9], all experiments with epidermal keratinocytes involve monolayers cultured in media, with no experiments conducted in the stratified or keratinized state[11, 14, 16, 18, 20, 22, 24, 26, 27]. Given that complex interactions occur between epidermal keratinocytes and dermal fibroblasts [29–35] and that epidermal keratinocytes are stratified and keratinized in vivo, these experimental methods are unlikely to precisely reproduce in vivo phenomena. Therefore, we have designed an experimental system wherein stretch stimuli are applied to human skin equivalents (HSEs) created by coculturing of these two types of cells as a means to perform experiments in settings closer to the in vivo conditions. HSEs are based on a 3-dimensional cultured-skin model developed using rat skin cells by Bell et al [36]. and have been used in various assays such as in vitro drug safety tests [37] and percutaneous absorption tests [38]. In this study, we developed a system allowing stretch stimulation of HSEs during their formation, and we analyzed the effects of stretching on keratinization of epidermal keratinocytes and on the basement membrane between the epidermal layer and the dermic layer. These phenomena could not be observed by means of conventional methods.

## Materials and Methods

### Cell culture

Normal human dermal fibroblasts (NB1RGB) and normal human epidermal keratinocytes (NHEK) were purchased from Riken Cell Bank (Tsukuba, Japan) and KURABO Industries (Osaka, Japan), respectively. NB1RGB cells were subcultured in the MEM-α medium containing 10% FBS (Wako Pure Chemical Industries, Osaka, Japan), and subcultures between 3–8 passages were used. NHEK cells were subcultured in the serum-free keratinocyte growth medium HuMedia-KG2 (KURABO Industries), and subcultures between 2–4 passages were used in experiments.

### Stretching chamber

**Fig 1:**
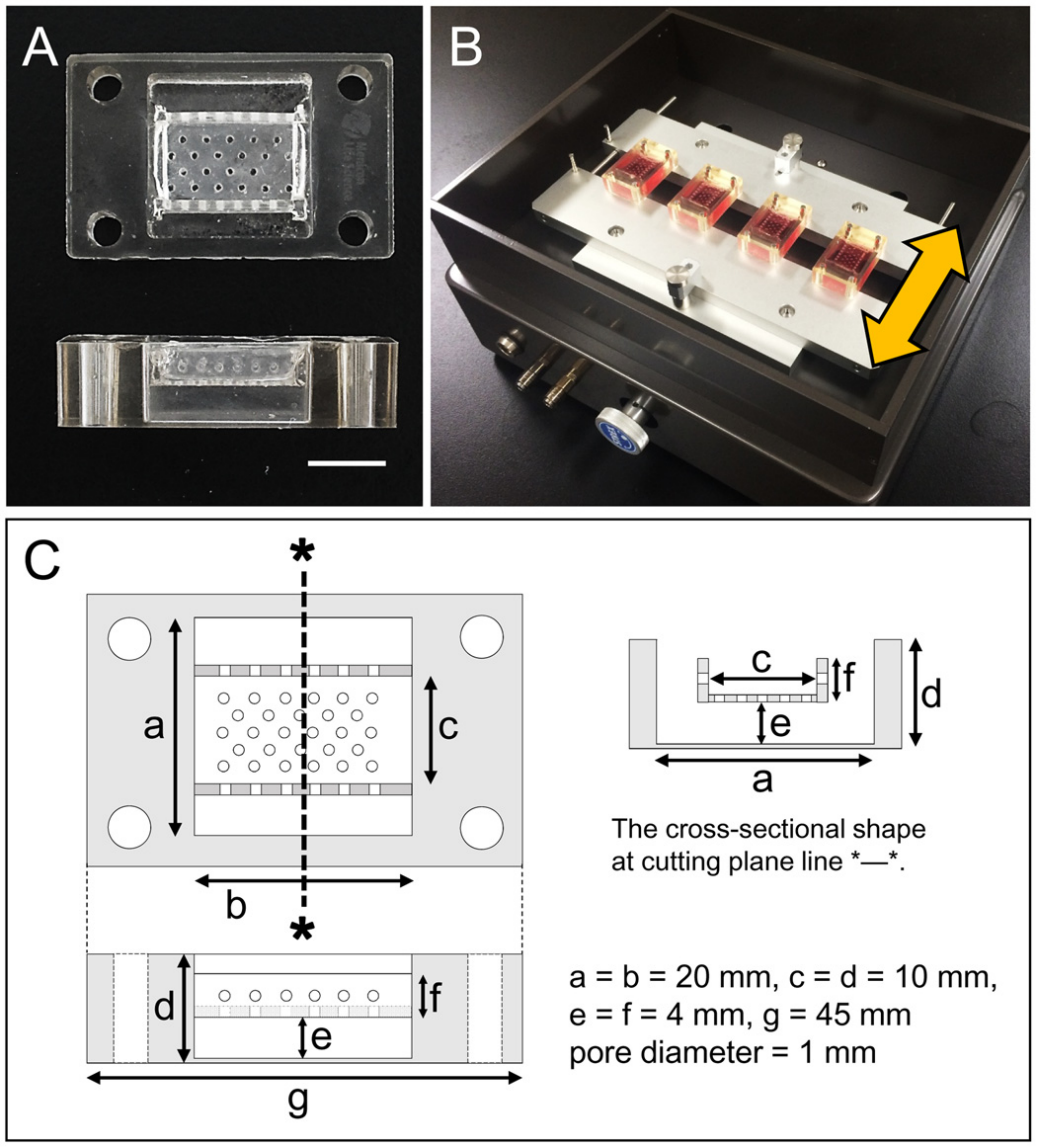
Stretching chamber and stretch device. (A) Over and side view of the chamber. Scale bar = 10 mm. (B) Overhead view of the stretch device. Yellow arrow shows the stretching direction. (C) Schema of the chamber.

In this experiment, we devised a stretch chamber for applying a mechanical stretch to HSEs while they form. Three porous silicone sheets (pore diameter, 1 mm) were attached to the inside of a conventional silicone chamber (Menicon, Aichi, Japan) to form a gutter-like shape. A silicone resin (TSE3032; GE Toshiba Silicones, Tokyo, Japan) was used as an adhesive. The “gutter” was placed such that its bottom was located 4 mm above the bottom of the chamber (Fig 1A and 1C). Prior to use, the chamber was subjected to a 90-seconds plasma treatment with a vacuum plasma apparatus (YHS-R; SAKIGAKE-Semiconductor, Kyoto, Japan) to impart hydrophilicity to the silicone sheet surface.

### Human skin equivalents (HSEs)

**Fig 2:**
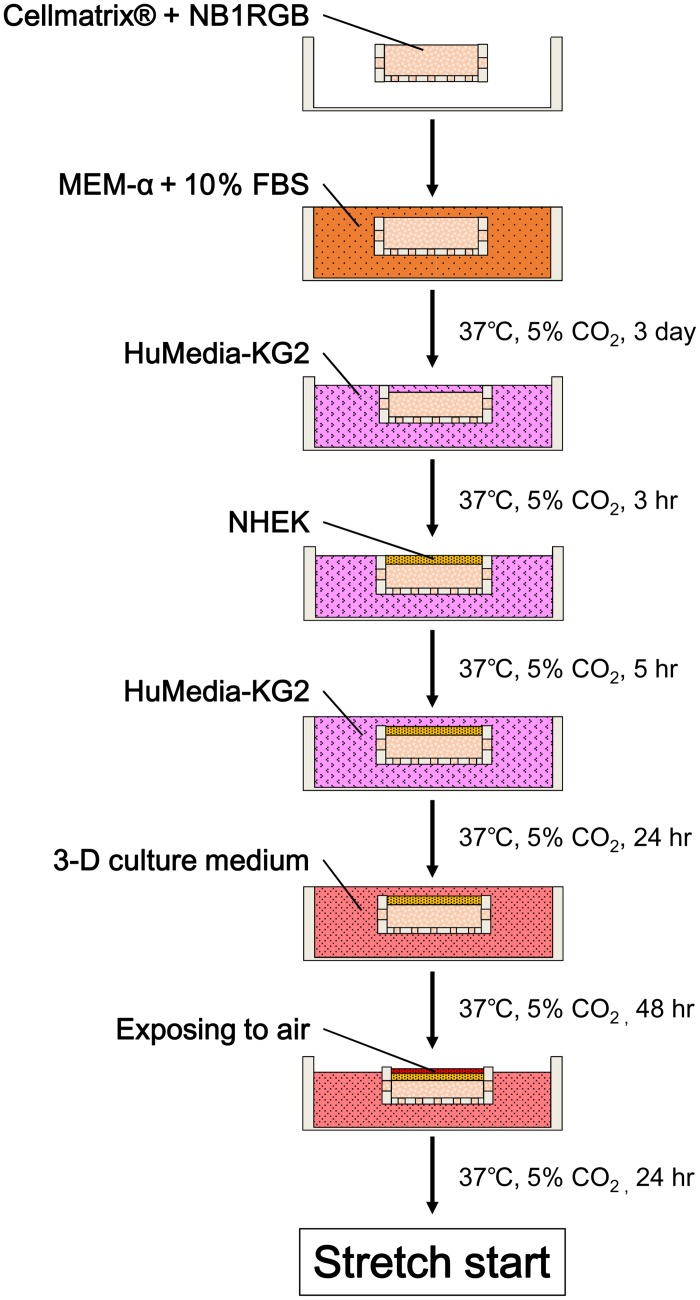
Procedure for establishing HSEs.

Six hundred microliters of a 0.2% solution of type 1 collagen (Cellmatrix^®^; Nitta Gelatin, Osaka, Japan) containing NB1RGB cells (1.0 × 10^5^/mL) was injected into the gutter. After letting it settle in a CO_2_ incubator at 37°C for 30 minutes for gelation, we filled the chamber with the MEM-α medium supplemented with 10% FBS and then incubated the chamber in 5% CO_2_ at 37°C for 3 days. After removing MEM-α from the chamber by aspiration, HuMedia-KG2 was injected and allowed to incubate for 3 hours to replace the medium in the gel. A 100-μL suspension of NHEK cells (1.0 × 10^6^ cells/mL) in HuMedia-KG2 was poured onto the collagen gel and incubated without agitation for 5 hours to allow the adherence of NHEK cells to the collagen gel. Subsequently, Humedia-KG2 was injected to fill the chamber to its upper edge. After 24-hours incubation, the medium in the chamber was replaced with a 1:1 mixture of the MEM-α medium and HuMedia-KG2 supplemented with 5% FBS, 1.8 mM Ca^2+^, and 50 μg/mL ascorbic acid (3-D culture medium). After 48-hours incubation, the amount of the medium in the chamber was reduced such that the skin model surface was exposed to the air and thereby keratinized. After incubating it for an additional 24 hours, we initiated stretch loading (Fig 2).

### Stretching HSEs

After preparing HSEs as described in Section 2.3, we mounted the chamber on a stretch device (STB-140; STREX, Osaka, Japan) (Fig 1B). To this model, uniaxial stretch stimuli were applied periodically (stretch rate 10%, stretch and return speed: 10%/sec, hold time: 30 seconds, waiting time before next stretch: 30 seconds) for 5 days (stretched sample: ST). HSEs that were prepared in parallel using the same chamber without stretch stimulation were used as a control (non-stretch sample: NST). In the course of these cultures, HSEs occasionally exfoliated from the silicone sheets and shrunk; these were discarded and not used for analysis.

### Histology and immunofluorescence staining

HSEs were fixed with 4% paraformaldehyde, embedded in paraffin, sectioned at 4.5 μm, and stained with hematoxylin and eosin (H&E).

For immunofluorescence (IF) staining, HSEs in an embedding agent (Tissue-Tek^®^ O.C.T. Compound; Sakura Finetek Japan, Tokyo, Japan) were frozen in liquid nitrogen and sectioned at 7 μm with a cryostat. Sections were incubated with the following primary antibodies: a mouse anti-involucrin monoclonal antibody (ab68; Abcam, Cambridge, UK, diluted 1:200), rabbit anti-type IV collagen polyclonal antibody (ab6586; Abcam, diluted 1:100), mouse anti-laminin 5 monoclonal antibody (ab102539; Abcam, diluted 1:500), and mouse anti-type VII collagen monoclonal antibody (NU-01-CO7; COSMO BIO, Tokyo, Japan, diluted 1:100). An Alexa Fluor 488–conjugated anti-mouse IgG antibody (A-11001; Thermo Fisher Scientific, MA, USA, diluted 1:200), Alexa Fluor 555–conjugated anti-rabbit IgG antibody (A-21428; Abcam, diluted 1:200) and Alexa Fluor 555–conjugated anti-mouse IgG antibody (A-21427; Abcam, diluted 1:200) were used as secondary antibodies. Nuclear counter-staining was performed using 4',6-diamidino-2-phenylindole (D-523; Dojindo, Kumamoto, Japan; diluted 1:1500). Fluorescent images were acquired using a box-type fluorescence microscope (FSX-100; Olympus, Tokyo, Japan).

### Transmission electron microscopy (TEM)

HSEs were prefixed with 2% paraformaldehyde containing 2% glutaraldehyde at 4°C overnight, and then postfixed with 1% osmium tetroxide at 4°C for 1 h. After dehydration using graded concentrations of ethanol (50–100%), the specimens were embedded in Epon 812 (Oken Shoji, Tokyo, Japan) and ultrathin sections (60–90 nm) were prepared on an ultramicrotome (EM UC6; Leica, Vienna, Austria). The sections were stained with 5% aqueous uranyl acetate and lead citrate and examined under a transmission electron microscope (H-7650; Hitachi, Tokyo, Japan) at 80 kV.

### Statistical analysis

Data from the microscopy analysis and from the immunoblotting assay of HSEs were expressed as mean ± SD. Differences in mean values between the NST and the ST group were assessed using Mann Whitney U test and were considered significant when p < 0.05. JMP 8 (SAS Institute, NC, USA) was used for data analysis.

## Results

### Stretch causes an increase in the thickness of the epidermal layer

**Fig 3:**
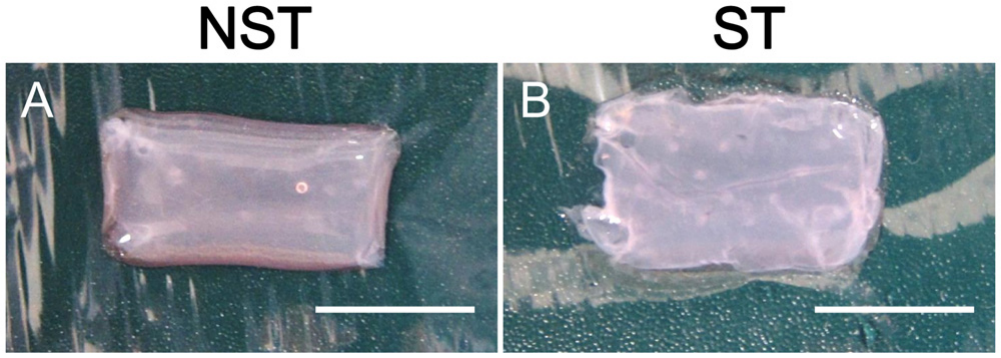
Macroscopic view of the harvested HSEs. (A) Non-stretch sample. (B) Stretched sample. Stretched sample had a thicker cuticle and reduced transparency compared with the non-stretch sample. Scale bar = 10 mm.

Based on macroscopic findings, the ST group had a thicker cuticle and reduced transparency compared with the NST group (Fig 3).

**Fig 4:**
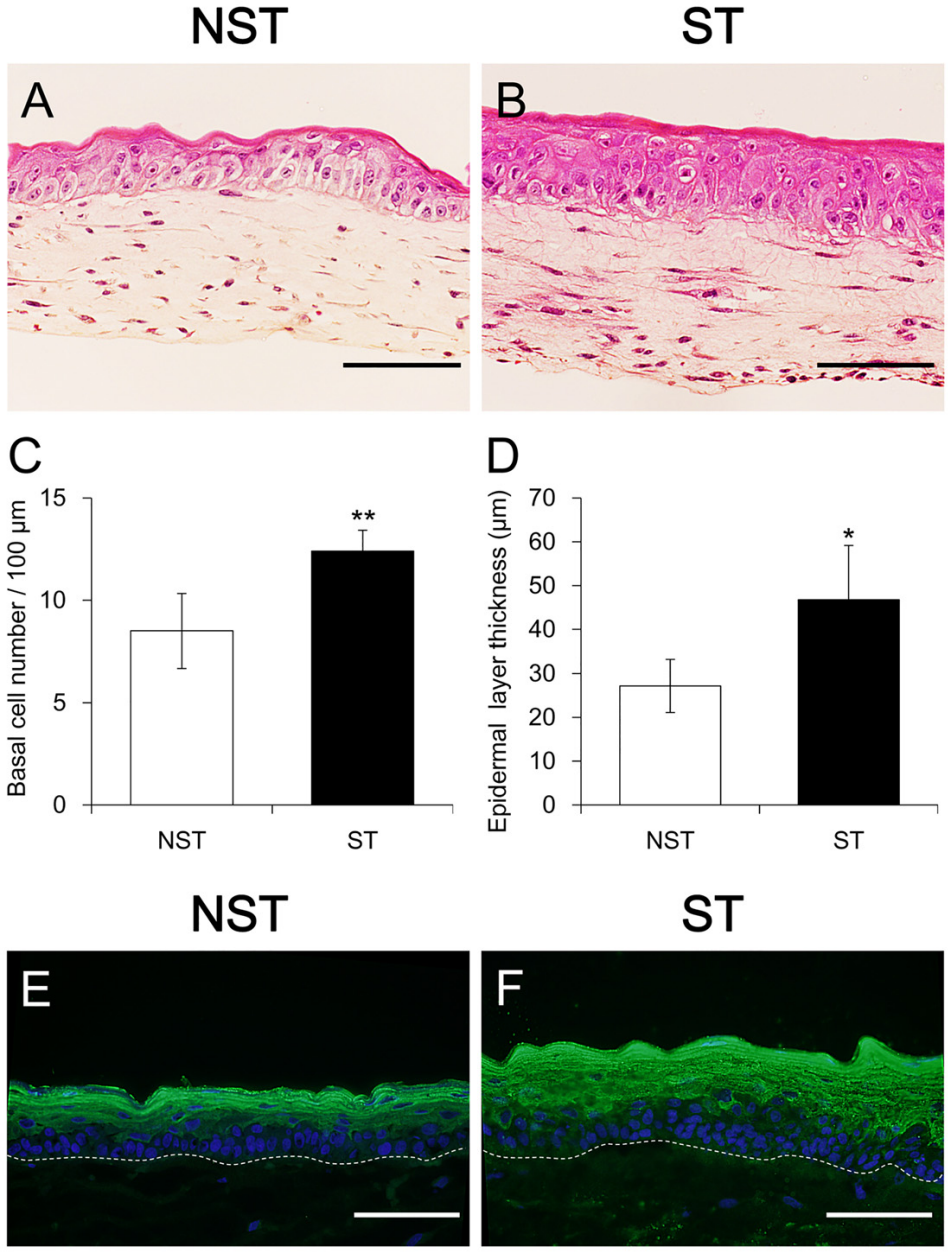
Histlogic and immunohistologic analysis of HSEs. (A) Hematoxylin and eosin staining of the non-stretch sample (B) and of the stretched sample. Scale bar = 100 μm. (C and D) The number of basal cells per 100 μm of a dermal—epidermal junction and the thickness of the epidermal keratinized layer showing a significant increase in the ST group. **p < 0.01 and *p < 0.05. (E and F) The expression of involurin was significantly increased in the ST group. Dotted lines indicate basement membrane. Scale bar = 100μm.

In H&E-stained sections (Fig 4A and 4B), the number of basal cells per 100 μm of a dermal—epidermal junction (mean of randomly picked five points) was 8.50 ± 1.83 cells in the NST group (mean ± SD, n = 6) and 12.40 ± 1.03 cells in the ST group (mean ± SD, n = 8), showing a significant increase in the ST group (p < 0.01) (Fig 4C). Furthermore, the thickness of the epidermal layer (mean of randomly chosen 10 points) was 27.2 ± 5.94 μm in the NST group (mean ± SD, n = 6) and 46.8 ± 12.4 μm in the ST group (mean ± SD, n = 8), thereby showing a significant increase in the ST group (p < 0.05) (Fig 4D).

Immunofluorescence staining of the sections for involucrin as a measure of the keratinization status revealed significantly increased expression of involucrin in the ST group (Fig 4E and 4F). These results suggest that stretch stimulation promotes keratinization of epidermal basal cells in HSEs and induces an increase in the thickness of the epidermal layer.

### Stretch increases the deposition of laminin 5, collagen IV/VII in the basal layer

**Fig 5:**
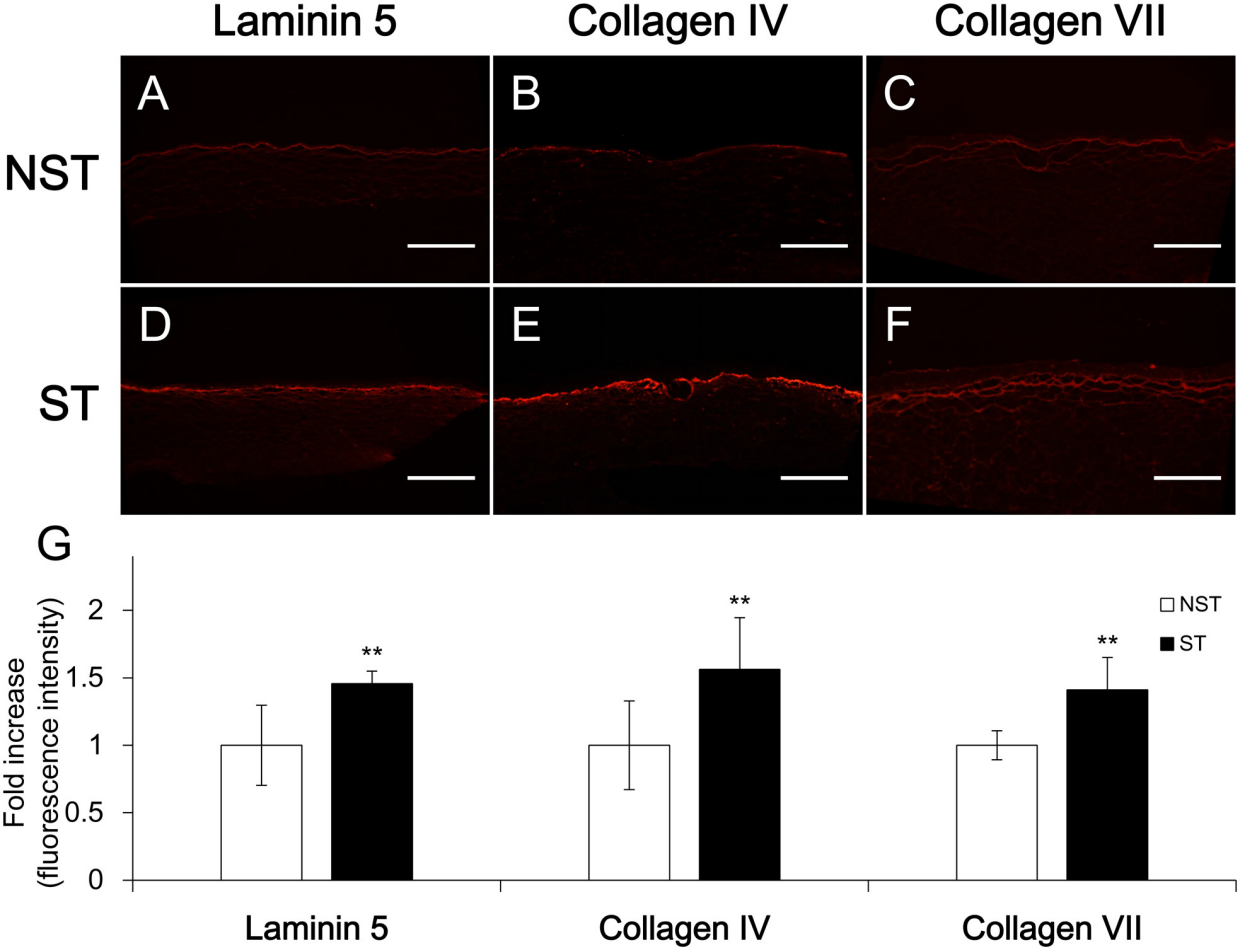
Laminin 5 and collagen IV/VII expression analysis of HSEs by immunofluorescence staining. (A, B and C) Non-stretch sample. (D, E and F) Stretched sample. Scale bar = 500 μm. (G) Fluorescence intensity of NST group was taken as control and adjusted to the 1 value. Each histogram bar represents the mean value of the normalized and adjusted fluorescence intensity. All three proteins of ST group were significantly greater compared with NST group. **p < 0.01.

In the tissue slices subjected to immunofluorescence staining for laminin 5 and collagen IV/VII (Fig 5A–5F), the fluorescence intensity in the basal membrane was measured at five randomly selected points, and the average value was used for comparison. In the ST group, the laminin 5 staining intensity was 1.45 ± 0.09 times (mean ± SD, n = 10) greater compared with the control basal value (NST: 1.00 ± 0.30, mean ± SD, n = 8). Intensity of collagen IV staining was 1.56 ± 0.38 times (mean ± SD, n = 11) greater compared with the control basal value (NST: 1.00 ± 0.33, mean ± SD, n = 10); intensity of collagen VII staining was 1.41 ± 0.24 times (mean ± SD, n = 7) greater compared with the control basal value (NST: 1.00 ± 0.11, mean ± SD, n = 6); and the differences were statistically significant (p < 0.01 for all 3; Fig 5G). This result suggests that the stretch stimulus acted on the skin cells in HSEs and promotes deposition of basement membrane proteins onto the basal layer.

### Stretch develops the formation of the basement membrane

**Fig 6:**
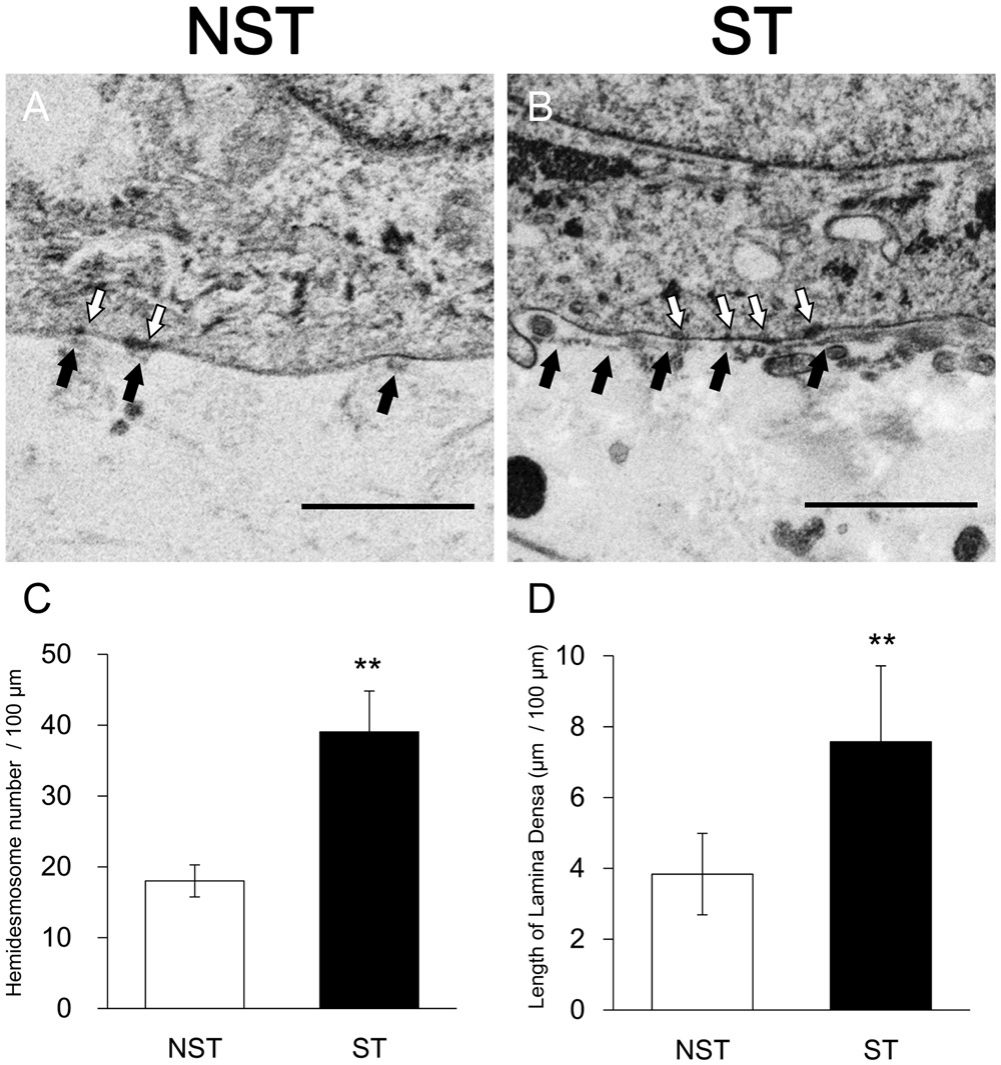
TEM images of HSEs. (A) Non-stretch sample. (B) Stretched sample. White arrow: hemidesmosome. Black arrow: lamina densa. Scale bar = 1 μm. (C, D) In the ST group, the length of lamina densa and the number of hemidesmosomes per 100 μm of a dermal—epidermal junction were significantly greater than in the NST group. **p < 0.01.

Electron micrographs of overlapping fields of basal area enlarged 4,000 times (Fig 6A and 6B) were printed out. The number of hemidesmosomes and the length of lamina densa of each focal area was counted and measured on the photographs, and the average number of hemidesmosomes and the length of lamina densa per 100 μm of the dermal-epidermal interface was calculated. The number of hemidesmosomes per 100 μm of the dermal-epidermal interface was 18.01 ± 2.26 in the NST group (mean ± SD, n = 5) and 39.01 ± 5.76 in the ST group (mean ± SD, n = 5), showing a significant increase in the ST group (p < 0.01) (Fig 6C). And the length of lamina densa per 100 μm of the dermal-epidermal interface was 3.84 ± 1.15 μm in the NST group (mean ± SD, n = 5) and 7.57 ± 2.15 μm in the ST group (mean ± SD, n = 5), thereby showing a significant increase in the ST group (p < 0.01) (Fig 6D). This TEM analysis revealed that stretch stimulation led to formation of more developed basal membrane structures.

## Discussion

According to previous reports, application of stretch stimulation to dermal fibroblasts triggers signal transduction from ECM into cells via phosphorylation of focal adhesion kinase (FAK) mediated by β1 integrin in the cell membrane [13], resulting in increased synthesis of ECM proteins such as collagen I and III and elastin [7, 19, 23], as well as increased production of protease inhibitors such as plasminogen activator inhibitor (PAI) and tissue inhibitor of metalloproteinase (TIMP) [23].

In epidermal keratinocytes, stretch stimulation is also transmitted into cells via β1 integrin and induces ERK phosphorylation [24], as is the case in dermal fibroblasts. Nonetheless, the pattern of β1 integrin redistribution on the cell membrane after application of stretch stimuli is similar to that of epidermal growth factor receptor (EGFR) [16]. The stretch signaling in epidermal keratinocytes is believed to involve interactions between β1 integrin and EGFR. In epidermal keratinocytes, stretch application promotes cellular adhesiveness [16], cell proliferation [22, 24, 26], and protein synthesis [26]. In normal human skin, keratinocytes proliferate in the basal layer and gradually migrate towards the surface, flattening out and becoming more differentiated towards the anuclear horny cells of the stratum corneum. At each stage of differentiation, keratinocytes express specific differentiation markers. Typically, keratinocytes in the basal layer express K14, a marker of proliferative status, whereas those in the spinous layers express K10, a marker of differentiation status, and those in the granular layers express involucrin, a marker of keratinization status [39]. Here, we analyzed the expression levels of these markers and found increased expression of involucrin. In contrast, K10 and K14 expression levels, indicating the differentiation and proliferative status, respectively, were not significantly altered, but showed a tendency to increase (data not shown).

There are various reports regarding the effects of basement membrane proteins on the basement membrane structure and epidermal layer formation. During preparation of HSEs, laminin 5, when added to the culture medium, reportedly promotes formation of lamina densa [40]. Incubation of the cells with inhibitors of proteolytic enzymes, matrix metalloproteinase (MMP), and plasminogen increases deposition of laminin 5 and collagen IV/VII on the basement membrane, leading to improvement of basement membrane structure as well as stratification and keratinization of the epidermal layer [41, 42]. In addition, there are reports about the use of a collagen IV sheet [43] and an amniotic membrane [44] as the basement membrane, and in both reports, differentiation of the epidermal layer is improved compared to conventional HSEs.

On the basis of these findings along with our study, it may be possible that stretch stimulation of HSEs is first transmitted to epidermal keratinocytes attached to the dermal layer; then cell proliferation, protein synthesis, and adhesiveness are enhanced; the signal is transmitted to dermal fibroblasts at the same time; and protein synthesis and protease inhibitor secretion are enhanced. Therefore, deposition of laminin 5, collagen IV, and collagen VII in the basal layer is increased, the basement membrane structure becomes more developed, and thereby keratinization of epidermal keratinocytes is enhanced further. The thickness of the keratinocyte layer is increased.

Dermal fibroblasts in the dermal layer are required for maintenance of the epidermal layer; without them stratification of the epidermis does not proceed properly. Complex interactions between epidermal keratinocytes and dermal fibroblasts are intimately involved in this phenomenon, and it is known that IL-1α from epidermal keratinocytes acts on dermal fibroblasts to regulate production of keratinocyte growth factor (KGF) and granulocyte macrophage colony-stimulating factor (GM-CSF). Consequently, the epidermal layer is maintained in an appropriate condition [33]. Although we did not measure cytokines secreted by the cells in the present experiments, it is conceivable that stretch stimuli applied to the skin affect cytokine-mediated interactions between epidermal keratinocytes and dermal fibroblasts to some extent. This effect may be related to delayed wound healing and high incidence of hypertrophic scars in skin regions exposed to stretching.

## Conclusions

In this study, we developed systems that enable application of stretch stimuli to HSEs during formation. Consequently, we found that in the ST group, the epidermal layer is thicker than NST group. Furthermore, synthesis of basement membrane proteins and deposition in the basal layer are increased; therefore, a more developed basement membrane is formed. Further research using this system may elucidate effects of stretching on skin properties and wound healing. In addition, application to an in vitro model of a hypertrophic scar is also expected.
